# Opioid Receptor Expression in Colorectal Cancer: A Nested Matched Case-Control Study

**DOI:** 10.3389/fonc.2022.801714

**Published:** 2022-04-06

**Authors:** Amparo Belltall, Guido Mazzinari, Iris Garrido-Cano, Francisco Giner, Anabel Marqués Marí, Pilar Eroles, María Pilar Argente-Navarro, Juan Pablo Cata, Oscar Diaz-Cambronero

**Affiliations:** ^1^Research Group in Perioperative Medicine, Hospital Universitario y Politécnico la Fe, Valencia, Spain; ^2^Department of Anaesthesiology, Hospital Universitario y Politécnico la Fe, Valencia, Spain; ^3^Euro-Periscope, Onco-Anaesthesiology Research Group (RG) of European Society of Anaesthesiology and Intensive Care, Brussels, Belgium; ^4^INCLIVA Biomedical Research Institute, Valencia, Spain; ^5^Department of Medical Oncology, University of Valencia INCLIVA-Hospital Clínico de Valencia-Centro de Investigación Biomédica en Red - Oncología (CIBERONC), Valencia, Spain; ^6^Department of Pathology, Hospital Universitario y Politécnico la Fe, Valencia, Spain; ^7^Department of Anesthesiology and Perioperative Medicine, The University of Texas MD Anderson Cancer Center, Houston, TX, United States; ^8^Anesthesia and Surgical Oncology Research Group, The University of Texas MD Anderson Cancer Center, Houston, TX, United States

**Keywords:** neoplasm, tumor, cancer, immunohistochemistry, opioid receptors, perioperative opioid, cancer, surgery

## Abstract

**Background:**

There is growing interest in the possible effect of perioperative anesthetic management on the growth and spread of cancer. The impact of perioperative use of opioids on cancer recurrence remains controversial and an assessment cannot yet be established based on current publications. This study aimed to assess the differential expression of opioid receptors between healthy and tumor tissues in patients with stage II and III colorectal cancer undergoing elective surgery by immunohistochemistry (IHC).

**Methods:**

Propensity–score matched case–control study nested in a retrospective cohort of patients with stage II or III colorectal. The primary endpoint was the difference in µ–opioid receptor (MOR) expression measured by IHC between tumor and healthy tissue in subject with or without recurrence. Secondary endpoints were to evaluate the differences in Opioid Growth Factor Receptor (OGFR), cyclic adenosine monophosphate (cAMP) production and protein kinase A (PKA) in the matched sample and from a from samples of colorectal cancer stored in the Cancer Genome Atlas (TCGA) and Genotype Tissue Expression Project (GTEx).

**Results:**

There was a significant difference in MOR receptor (median 3 [intequartile range IQR: 1–3] and 0 [IQR: 0–2], P<0.001) and OGFR receptor (median 6 [IQR: 5–6] and 2 [IQR: 1–2], P<0.001) in tumor and control tissue respectively. However, there were no significant differences in cAMP nor PKA expression between both types of tissues and in expression in any of the analyzed variables by recurrence status. The MOR and OGFR expression data from TCGA database were similar to our sample size data with lower expression of MOR and higher expression of OGFR in tumoural samples with a skewed distribution for MOR expression in tumor tissue both in patients with and without recurrence.

**Conclusion:**

In patients with stage II and III colorectal cancer, overall expression of MOR and OGFR was significantly increased but was not different between previously matched patients with or without recurrence. No differences were found in the analyzed metabolic pathway of cAMP–PKA: These results were confirmed by an *in silico* analysis of samples from the TCGA–GTEx database.

## Introduction

Opioids are potent analgesics indicated for moderate-to-severe pain management in patients undergoing cancer surgery. Opioids have several cellular targets such as µ, κ and δ (MOR, KOR, and DOR, respectively) and opioid growth factor (OGFR) receptors (1–3). Preclinical studies suggest that opioids could promote direct tumor growth, angiogenesis, metastasis, and cellular and humoral immunosuppression (4–6). Among the proposed mechanisms for these pro–tumoral effects is the activation of MOR, which has been shown to be overexpressed by tumor cells in colorectal cancer (7–9).

While guidelines exist for evaluating the expression of receptors in cancer cells (10), there is no validated consensus for immunohistochemistry (IHC) staining for opioid receptors. Typically, MOR expression is determined by using IHC and measuring staining intensity on a grading scale. Some variability depending on the type of sample and reagents is documented in studies assessing MOR expression in various types of cancers (8, 9, 11–18). Furthermore, IHC can have a considerable intraobserver and interobserver (19, 20) and can be only moderately correlated with quantitative methods such as the real-time quantitative reverse transcription-polymerase chain reaction (RT–qPCR) that do not require visual assessment and can be automated (19, 21–23).

As for the other opioid receptor targets, the OGFR has shown inhibitory effects in tumor growth (3), while the role of DOR and KOR are even more controversial with data showing both activating (24) and suppressing effects (25) which can be explained by a different profile of receptor expression (16). In addition, activated opioid receptors trigger several intracellular responses that are responsible for their divergent pharmacological outcomes. For instance, many morphine analogs target MOR *via* two distinct signaling pathways independently associated with analgesic properties and unwanted side effects (26). Analgesia is achieved through a classical G-protein pathway that suppresses neuronal excitability and promotes neuronal hyperpolarization by regulating intracellular cyclic adenosine monophosphate (cAMP) production and protein kinase A (PKA) activity (27).

This study aimed to assess by IHC the difference in opioid receptors expression between healthy and tumor tissues in patients with stage II and III colorectal cancer undergoing elective surgery. Our primary objective was to determine the difference in MOR expression measured by IHC between tumor and healthy tissue in patients who experience tumor recurrence versus patients who do not suffer it. Secondary objectives were to evaluate the differences in OGFR receptor, cAMP, and PKA expression and to evaluate the difference in expression of MOR and OGFR between tumor and healthy tissues from samples of colorectal cancer stored in the Cancer Genome Atlas (TCGA) and Genotype Tissue Expression Project (GTEx).

## Methods

This was a propensity score matched case-control study nested in a retrospective cohort of patients with stage II or III colorectal cancer undergoing elective surgery from an investigator-initiated single–center study carried out at the University and Polytechnic Hospital la Fe in Valencia, Spain, which was conducted after Institutional Review Board approval (#Morocco, March 2018) and registration at clinicaltrials.gov (NCT03601351) and is published elsewhere (9).

### Study Population

The original study included 174 patients who underwent scheduled colorectal surgery for stage II and III primary colorectal cancer from January 2010 to December 2014 and excluded patients with stage I or IV colorectal cancer, those undergoing emergency or non–oncological surgery, and those with poor quality histological samples. This cohort of patients was followed for five years starting from the day of surgery, and the primary tumor recurrence was recorded. From this cohort, we randomly sampled 27 patients with recurrence and matched them in a 1:1 ratio with the optimal method and a caliper of < 0.1 without replacement with subjects without recurrence. The variables used for matching were: Dukes stage, number of affected lymph nodes, and tumoral tissue differentiation. Only subjects with stage II or III cancer and good or moderate tissue differentiation were included in the analysis.

### Laboratory Methods

To grade the IHC we used the same scale as previously described. (9) Antibodies against OGFR (Proteintech), MOR1 (ORMU) (Abcam, Cambridge, United Kingdom), cAMP(Millipore, Merck, Burlington, Massachusetts, United States) and PKA (Cell Signaling, Danvers, Massachusetts, United States) were used to measure the expression of each biomarker, in paraffin sections of colorectal adenocarcinoma and adjacent normal tissues (control tissue). All antibodies were used following the company instructions. We used different dilutions for OGFR (1:1000), ORMU (1:300), cAMP (1:200), and PKA (1:200), according to our previous tests on different tissue controls. The slides were stained for 10 minutes with 3,3′-diaminobenzidine chromogen and counterstained for ten minutes with hematoxylin.

The quantification of MOR, OGFR, cAMP, and PKA expression in study samples was done by microscopic evaluation of immunoreactivity carried out by one experienced pathologist. Immunostaining control was previously tested successfully in central nervous system tissue sample without MOR expression. After the first immunostaining reading, the same pathologist conducted a second assessment to minimize interindividual variability. If good concordance was observed, the final reading was used for analysis; otherwise, a median score was calculated. To grade the IHC we used the same scale as previously described (9). Immunostaining was read in a semi-quantitative manner. Positive staining was defined as a sample showing brown signals in the cell cytoplasm, nucleus, or membrane. The staining intensity was scored as 0 (no staining), 1 (weakly stained), 2 (moderately stained), or 3 (strongly stained). The percentage of cell positivity was scored as 0 (< 5%, negative), 1 (5%-25%, sporadic), 2 (25%-50%, focal), or 3 (>50%, diffuse). MOR expression was scored by adding the intensity staining scores and the percentage area positively stained, producing a total range from 0 to 6.

### Gene Expression Analysis

To assess the expression of the opioid receptor at genomic levels, we used RNA–sequencing (RNA–seq) data from the TCGA and GTEx repositories. These are big repositories containing genetic data from cancer tissues and healthy individuals, respectively. However, these large databases are not directly comparable as differences in samples processing, and analysis pipeline across the different studies whose data are stored in the databases make an integrative analysis difficult. Thus, we used normalized data from a publicly available database (https://figshare.com/articles/dataset/Data_record_1/5330539). In addition, this study removed batch effects through an *ad hoc* developed pipeline (28). The details of the used code are available at: https://github.com/mskcc/RNAseqDB and https://github.com/mskcc/RNAseqDB/blob/master/README.md. RNA–seq expression data were log–transformed for the analysis. We selected stage II and III samples from the retrieved cases.

### Statistical Analysis

Since the purpose of the analysis was exploring physiological hypotheses, we did not specify any *a priori* effect size and performed analysis without formal sample size calculations.

Quantitative variables are expressed according to the distribution recorded as mean and standard deviation (SD) or median and interquartile range [25^th^ – 75^th^ percentile], and categorical variables as proportions and counts. We checked the normality of each variable’s distribution by applying the Shapiro-Wilk test and examining quantile–quantile plots.

The overall and by recurrence difference in MOR, OGFR, cAMP and PKA expression between tumor and healthy tissues was evaluated using the Wilcoxon signed rank test for paired samples. In addition, the difference between MOR and OGFR between subjects with or without recurrence in the TCGA database was performed by the Wilcoxon rank sum test.

Statistical significance was set at two-tailed P < 0.05. Bonferroni multiple comparison correction was carried out. No imputation routine of missing values was performed. The statistical analysis was performed using the statistical software R (version 4.0.1, The R Foundation for Statistical Computing, www.r-project.org).

## Results

**Figure 1:**
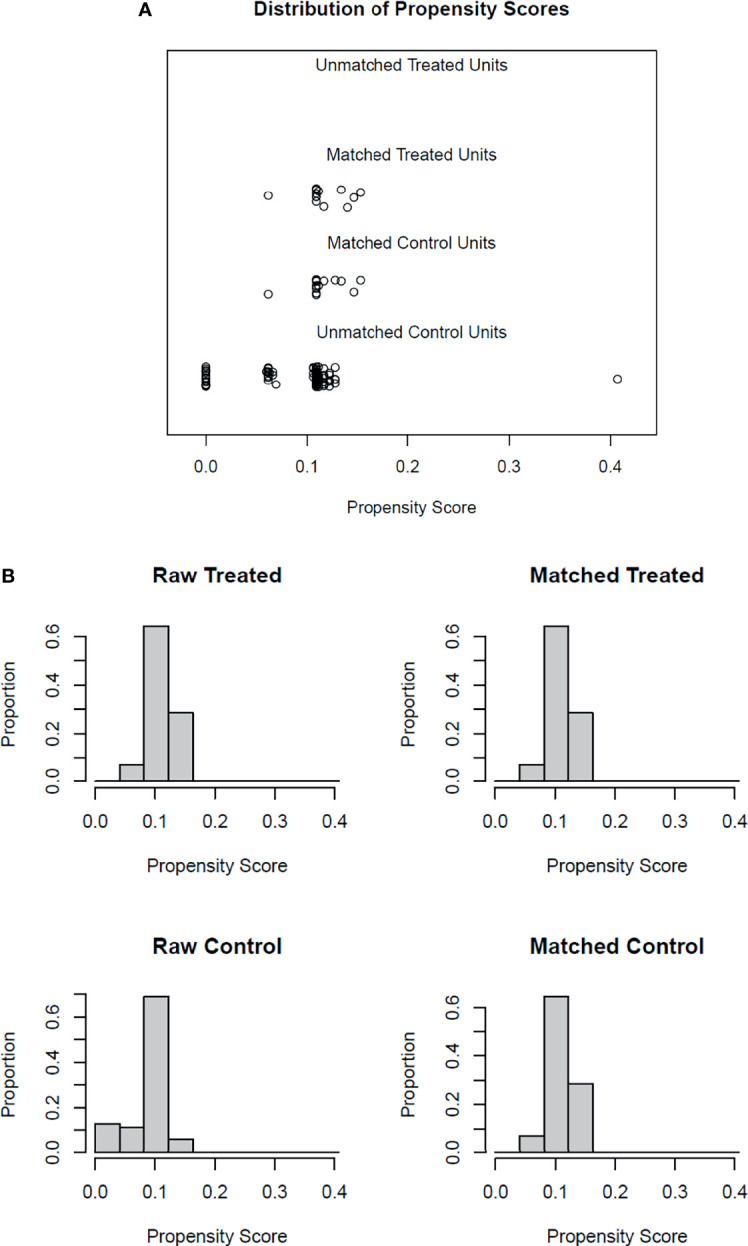
Propensity score matching diagnostic plots. Panel **(A)** jitter plot of propensity scores. The middle lines show the close match between the randomly selected treatment units and the matched control units. The bottom line shows the unmatched control units not included in the analysis. Panel **(B)** Histogram distribution before and after the matching process.

**Figure 2:**
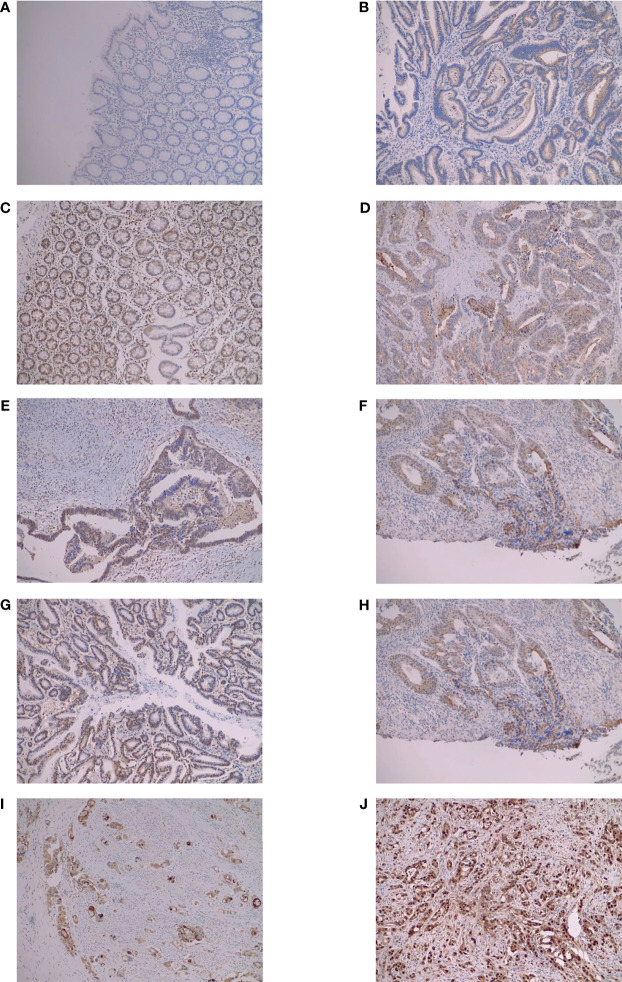
Immunohistochemical staining examples to describe scoring. All pictures are at 10X magnification. **(A)** Score 0 control cAMP; **(B)** score 1 tumor cAMP; **(C)** score 2 tumor OGFR; **(D)** score 3 tumor MOR; **(E)** score 4 tumor OGFR; **(F)** score 4 tumor MOR; **(G)** score 5 tumor OGFR; **(H)** score 5 tumor OGFR; **(I)** score 6 tumor MOR; **(J)** score 6 tumor OGFR. MOR, µ opioid receptor; OGFR, opioid growth factor receptor; cAMP, cyclic adenosine monophosphate.

We analyzed 27 subjects, 13 with and 14 without recurrence, satisfactorily matched for the preselected variables (i.e. Dukes stage, number of affected lymph nodes, and tumor tissue differentiation) **(** **Figure 1****)**. Some examples of IHC staining are shown in **Figure 2** to provide a graphical depiction of staining intensities. The concordance between readings was good.

**Figure 3:**
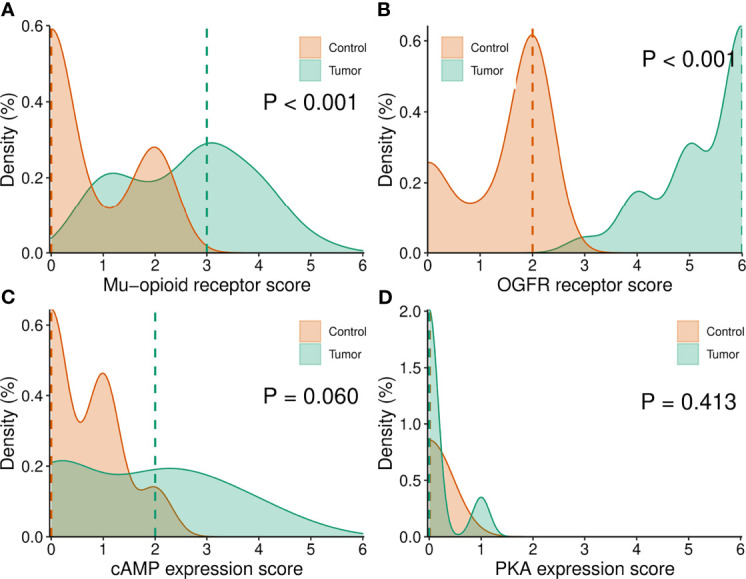
**(A–D)** Density plots of MOR, OGFR, cAMP and PKA expression determined by IHC by type of tumor. MOR, µ opioid receptor; OGFR, opioid growth factor receptor; cAMP, cyclic adenosine monophosphate; PKA, protein kinase A.

The distribution density plots by tissue type, i.e., control versus tumor, for MOR, OGFR, cAMP, and PKA are reported in **Figure 3**. There was a significant difference between control and tumor tissue in MOR and OGFR receptors, with higher expression levels in the tumor tissue. However, there were no significant differences in cAMP nor PKA expression between both types of tissues.

**Figure 4:**
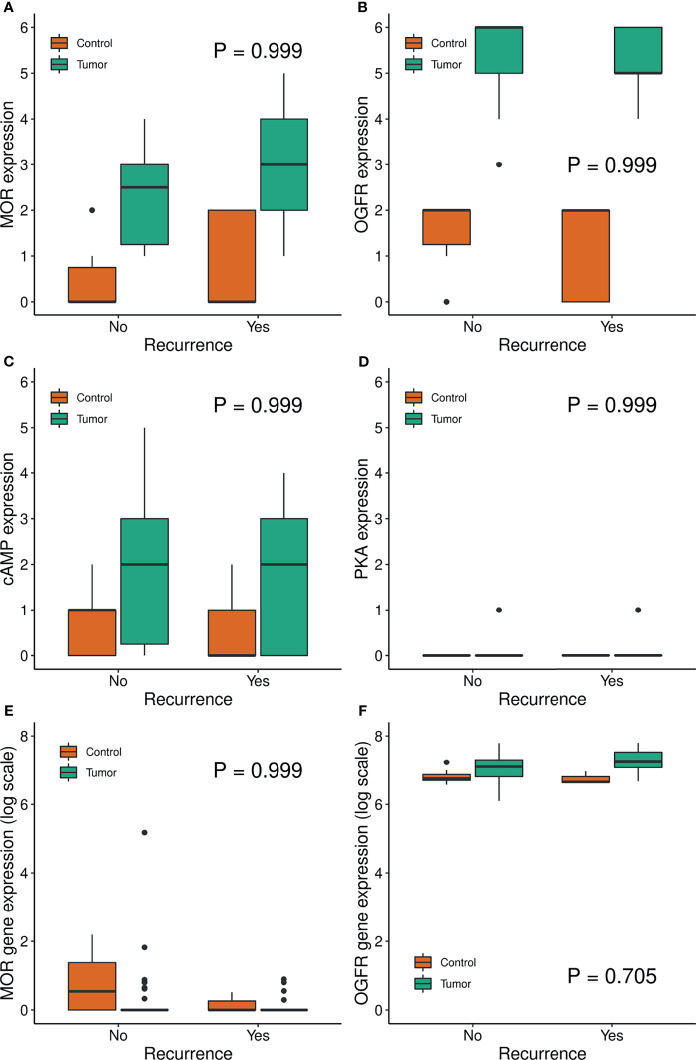
Boxplot of MOR, OGFR, cAMP, PKA expression by recurrence group. Panels **(A–D)** show results from IHC staining from the nested matched case-control sample. Panels **(E, F)** show gene expression from the TCGA and GTEx repositories.

Baseline characteristics and expression levels by tumor recurrence and tissue types are reported in **Table 1** and **Figure 4**. There were no significant differences in expression in any of the analyzed variables by recurrence status (**Figure 4**). The MOR and OGFR expression data from TCGA database were similar to our sample size data with low expression of MOR and higher for OGFR with a skewed distribution for MOR expression having values hovering towards 0 with few extreme outliers in tumor tissue both in patients with and without recurrence (**Table 1**).

**Table 1:** Sample baseline characteristics, receptors, and metabolic pathway expression.

Nested case-control sample			
	Recurrence		P value
	No (N = 14)	Yes (N =13)	
**Stage = III** % (N)	42.9 (6/14)	38.5 (5/13)	0.999
**Tumor differentiation = (moderate) % (N)**	92.9 (13/14)	92.3 (12/13)	0.999
**Lymph node affected (N)**	0 [0 – 3]	0 [0 – 2]	0.870
**MOR expression**			0.999*
Control	0 [0 – 1]	0 [0 – 2]	
Tumor	2 [1 – 3]	3 [2 – 4]	
**OGFR expression**			0.999*
Control	2 [1 – 2]	2 [0 – 2]	
Tumor	6 [5 – 6]	5 [5 – 6]	
**MOR expression**			0.999*
Control	0 [0 – 1]	0 [0 – 2]	
Tumor	2 [1 – 3]	3 [2 – 4]	
**cAMP expression**			0.999*
Control	1 [0 – 1]	0 [0 – 1]	
Tumor	2 [0 – 3]	2 [0 – 3]	
**PKA expression**			0.999*
Control	0 [0 – 0]	0 [0 – 0]	
Tumor	0 [0 – 0]	0 [0 – 0]	
**TCGA sample**			
	**Recurrence**		P value
	**No** (N = 89)	**Yes** (N= 20)
**MOR gene expression** (Log scale)			0.999**
Control (N = 16)	0.5 [0 – 1.3]	0 [0 – 0.2]	
Tumor (N = 93)	0 [0 – 0]	0 [0 – 0]	
**OGFR expression** (Log scale)			0.705**
Control (N = 16)	6.7 [6.7 – 6.9]	6.6 [6.6 – 6.8]	
Tumor (N = 93)	7.1 [6.8 – 7.3]	7.2 [7.1 – 7.5]	

*The Wilcoxon singed rank test is performed on the difference in expression between control and tumor tissue in subject with or without recurrence. *The wilconos rank sum test is performed on the difference in overall expression in subject with or without recurrence. ** The Mann-Whitney test is performed on the difference in overall expression in subject with or without recurrence. MOR, µ opioid receptor; OGFR, opioid growth factor receptor; cAMP, cyclic adenosine monophosphate; PKA, protein kinase A.

## Discussion

In this work, we investigated the association between MOR and OGFR receptor and the cAMP–PKA axis in colorectal cancer recurrence. Findings can be summarized as follows; first, the overall expression of MOR and OGFR receptor was significantly increased in colorectal cancer samples compared to paired control samples as assessed by IHC. Second, we did not find significant cAMP–PKA in colorectal cancer samples compared to paired control samples as assessed by IHC. Third, when we analyzed a sample of cases matched for relevant oncological features there were no differences between tumor and control tissue for receptor expression and secondary messengers. Lastly, these results were confirmed by an *in silico* analysis of samples from the TCGA–GTEx database.

To our knowledge, this is the first study evaluating how opioid receptor expression translates at the cellular level. Second, to minimize significant biases, we controlled the confounders by matching cases of recurrence with a similar sample of patients without recurrences. And third, we analyzed normalized data from large publicly available datasets to further corroborate our hypothesis and results from our retrospective single-center cohort of patients.

While we found a significantly higher MOR and OGFR expression in tumor tissue samples, we did not detect differences in expression of the receptors between subjects with and without recurrence in the matched analysis. The higher expression of MOR in tumor tissue is in line with previous findings from other authors that assessed such expression in different tumor types such as gastric (13), liver (15), esophagus (12), prostate (17), pancreas (11), lung (12), laryngeal (18), and colorectal cancer (9) as well as in cancer cell lines (8). Although most studies focused on the MOR receptor, more recent findings broadened the spectrum to other opioid receptors such as OGFR, suggesting that specific expression profiles may be behind an oncogenic propensity (16). For instance, OGFR has been linked to decreased cell proliferation in lung carcinoma (3) and breast cancer (29), and indeed, we did find that OGFR was overexpressed in our cancer samples. The rationale behind studying different molecular targets of opioid drugs is that a different balance between those exerting a protumor and antitumor effect can ultimately lead to a different modulating effect. In addition, other receptors such as the σ receptor (SR) have been shown to have an induction effect on MOR and DOR, although not technically an opioid receptor (30). Following and expanding on this concept it would be interesting to assess the entire roster of opioid receptors since there are seven known (i.e. MOR, DOR, KOR, SR, and ϵ, ζ, and λ opioid receptors), or to investigate the role of the different receptor subtypes. For instance, MOR type 1, which is the most studied subtype, is a well-known member of this receptor family with up to ten different variants already identified, although it is unclear if a different action can be attributed solely to a specific subtype (31).

The clinical significance of opioid receptors on long-term oncologic outcomes has been a subject of intense research in the last few years. A vast number of studies found an association between increased receptor expression and decreased disease-free survival (12, 15, 17, 18), while others did not find it (9, 14). Furthermore, more recent trials assessing several receptors found a diverging receptor expression layout with lower MOR and TLR4 but increased OGFR, KOR, and DOR expression and a protective effect of opioid administration on recurrence free survival (16). This protective effect confirmed a previous study that evaluated opioid administration without receptor expression assessment (32). It can be argued that to advance our knowledge of the effect of opioids on long-term oncologic outcomes, we must explore the entire molecular target profile and its interaction with opioid drugs administration in the perioperative period, even considering genetic variants (33).

Interestingly, while we found no differences between tumor and control tissue expression of MOR and OGFR in the TCGA–GTEx sample analysis, we observed a skewed distribution, which is even more remarkable given that the distribution is Log–transformed. Typically, whole tumor biopsies are used for qRT-PCR or RNA-seq analysis, limiting the ability to differentiate specific cell gene expression in various cell types. Whole tumor analysis may not provide sufficient resolution to identify changes in tissue sub-compartments. The assigning expressed genes could be confounded when gross extracts are used as mRNA source. Therefore, isolating individual cells or specific cell types from tissue sections will allow accurate detection of gene expression in that population. Altogether, this highlights the importance of tissue composition in data generation and the need to correctly define the extraction source to compare different experiments. The method of laser-capture microdissection (LCM) is an option to procure subpopulations of tissue cells under direct microscopic visualization to use in the following procedures (34, 35). These methodological issues are well documented in the literature, but there is no established standard yet (36).

Opioid receptors are G–coupled proteins and agonist-induced conformational changes favoring G-protein binding results in dissociation of its α-subunit from the β- and γ-subunit complex. The α-subunit inhibits adenylyl cyclase activity, reducing intracellular cAMP (26, 37, 38). Thus, cAMP and PKA levels measured by IHC may reflect the degree of MOR activation. However, this molecular pathway is not specific to opioid receptors (39). Also, opioid drugs also mediate their action *via* activation of the β-arrestin pathway, which regulates opioid receptor desensitization and internalization and is responsible for the opioid–mediated undesirable effects (37, 40). Even if exploring the activation of MOR pathways can be a promising path to gain insights on the effect of opioids on cancer, the scope has to be probably expanded to other known pathways and probably even to oncological pathways as recent trials are starting to explore (33).

Several limitations must be highlighted. First, the study’s retrospective design and the small sample size the findings should be seen as hypothesis-generating. Also, the small sample size limited the number of confounders we could introduce in the matching process to not exceed the recommended variable to case ratio. In addition, we focused on a specific MOR expression; thus, the influence of polymorphisms, other cellular pathways such β-arrestins or cannabinoid receptors, and opioid antagonists administration cannot be evaluated (41–43). Second, our analysis is limited to a specific subset of patients, i.e., stage II and III colorectal cancer patients; thus, extrapolation to other populations should be done with caution. Also, the matched cohort is based on Dukes’ stage, and TCGA–GTEx analysis is based on TNM classification. Thus, although significant overlap is present, this can limit the comparability between samples. Fourth, we observed a higher albeit non–significant MOR expression in control samples in the TCGA–GTEx samples analysis, which can be due to unpaired samples reading. In addition, although IHC readings were performed in a blinded fashion and showed good agreement, a certain degree of subjectivity inherent to semiquantitative IHC assays cannot be ruled out.

To conclude, in patients with stage II and III colorectal cancer, overall expression of MOR and OGFR was significantly increased but was not different between previously matched patients with or without recurrence. These findings were confirmed in a similar cohort extracted from the TCGA and GTEx databases. No differences were found in the analyzed metabolic pathway of cAMP–PKA. Further studies are warranted to comprehensively assess both the molecular footprint and metabolic pathways to elucidate whether opioids and specific expression profiles can impact long-term oncologic outcomes.

## Data Availability Statement

The raw data supporting the conclusions of this article will be made available by the authors, without undue reservation.

## Ethics Statement

The studies involving human participants were reviewed and approved by Instituto de Investigación Sanitaria la Fe. The patients/participants provided their written informed consent to participate in this study.

## Author Contributions

AB: This author conceived the idea, helped with data acquisition, critical review of the content, and manuscript preparation. GM: This author conceived the idea, helped with data acquisition, critical review of the content, and manuscript preparation. IG-C: This author provided a critical review of the content, and helped with manuscript preparation. FG: This author carried out the immunohistochemistry readings, critical review of the content, and manuscript preparation. AM: This author provided a critical review of the content, and helped with manuscript preparation. PE: This author provided a critical review of the content, and helped with manuscript preparation. MA-N: This author provided a critical review of the content, and helped with manuscript preparation. JC: This author provided a critical review of the content, and helped with manuscript preparation. OD-C: This author provided a critical review of the content, and helped with manuscript preparation. All authors contributed to the article and approved the submitted version.

## Conflict of Interest

OD-C: Received payment for educational talks and scientific conferences from MSD (Merck Sharp & Dohme, Inc.).

The remaining authors declare that the research was conducted in the absence of any commercial or financial relationships that could be construed as a potential conflict of interest.

## Publisher’s Note

All claims expressed in this article are solely those of the authors and do not necessarily represent those of their affiliated organizations, or those of the publisher, the editors and the reviewers. Any product that may be evaluated in this article, or claim that may be made by its manufacturer, is not guaranteed or endorsed by the publisher.
